# Integrative prescreening in analysis of multiple cancer genomic studies

**DOI:** 10.1186/1471-2105-13-168

**Published:** 2012-07-16

**Authors:** Rui Song, Jian Huang, Shuangge Ma

**Affiliations:** 1 Department of Statistics, Colorado State University, Fort Collins, USA; 2 Department of Statistics and Actuarial Science, University of Iowa, Iowa City, USA; 3 School of Public Health, Yale University, New Haven, USA

## Abstract

**Background:**

In high throughput cancer genomic studies, results from the analysis of single datasets often suffer from a lack of reproducibility because of small sample sizes. Integrative analysis can effectively pool and analyze multiple datasets and provides a cost effective way to improve reproducibility. In integrative analysis, simultaneously analyzing all genes profiled may incur high computational cost. A computationally affordable remedy is prescreening, which fits marginal models, can be conducted in a parallel manner, and has low computational cost.

**Results:**

An integrative prescreening approach is developed for the analysis of multiple cancer genomic datasets. Simulation shows that the proposed integrative prescreening has better performance than alternatives, particularly including prescreening with individual datasets, an intensity approach and meta-analysis. We also analyze multiple microarray gene profiling studies on liver and pancreatic cancers using the proposed approach.

**Conclusions:**

The proposed integrative prescreening provides an effective way to reduce the dimensionality in cancer genomic studies. It can be coupled with existing analysis methods to identify cancer markers.

## Background

In cancer research, high-throughput profiling studies have been extensively conducted, searching for genomic markers whose mutations or defects may increase susceptibility to cancer. Cancer genomic data has the “large *d*, small *n*” characteristic. For example, a typical microarray gene profiling study measures the expressions of 10^4^ genes and a genome-wide association study measures 10^6^ SNPs on 10^1-3^ subjects. For simplicity of discussion, we focus on cancer gene profiling studies using microarrays and note that the proposed approach is also applicable to other omics (e.g. epigenetics or proteomics) studies and other diseases.

Results from analysis of individual cancer genomic datasets often suffer from a lack of reproducibility [1-3]. The unsatisfactory reproducibility is also obvious from our numerical study. Although there are multiple possible causes, the most important one is “small *n*” and hence lack of power of individual studies. An ideal solution is to conduct large-scale prospective studies, which are extremely time-consuming and expensive. Knudsen [4] shows that for many cancer traits and clinical outcomes, there are multiple studies sharing comparable designs. A cost-effective way to improve reproducibility is to pool data from multiple existing studies and increase statistical power.

In cancer genomic data analysis, “large *d*” leads to high computational cost, particularly when it is necessary to simultaneously analyze all genes profiled. With most existing analysis approaches, computational cost increases significantly, linearly or even exponentially, as the number of genes increases. Under some scenarios, the computational cost may even put a ceiling on the number of genes that can be analyzed. For example, the R package WGCNA, which conducts the weighted gene co-expression network analysis, can only analyze ≤ 4000 genes [5]. An effective solution to the computational problem caused by “large *d*” is to conduct prescreening, which has relatively low computational cost, prior to complex analysis, which has high computational cost. Prescreening can be classified as unsupervised or supervised. Unsupervised prescreening does not utilize information on the response variable. For example, gene expressions with severe missingness or small variances are removed from downstream analysis. In contrast, supervised prescreening uses the response variables and can be more informative. In the analysis of single datasets, supervised prescreening studies include [6], which conducts numerical study of prescreening based on eight different statistics. Huang and others [7] proposed prescreening using a bridge penalization approach. Fan and Lv [8] developed the SIS (Sure Independence Screening) approach. Chen and Chen [9] proposed a tournament prescreening approach. Tibshirani [10] used a Lasso penalization approach for prescreening under the Cox model.

In this article, we investigate prescreening with multiple cancer genomic datasets sharing comparable designs. With multiple datasets, one possibility is to first conduct prescreening with each dataset separately. Meta-analysis can then be conducted to combine results from multiple datasets. Because of small sample sizes, prescreening results with individual datasets can be unsatisfactory. Meta-analysis cannot generate superior results using inferior inputs. Another possibility is to adopt intensity approaches, which transform gene expressions, combine multiple datasets, and conduct prescreening as if they were from a single study. Intensity approaches demand the full comparability of transformed gene expressions from different studies (platforms), which is still questionable. In addition, they need to be conducted on a case-by-case basis.

The goal of this study is to develop a practically useful prescreening approach for integrative analysis of multiple cancer genomic datasets. The proposed approach shares similar spirit with existing prescreening approaches [6-8,11]. Instead of fitting one model with *d* genes, *d* marginal models are fitted with only one gene in each model. A ranking statistic measuring marginal significance is computed in each marginal model, and only genes with statistics larger than a cutoff are analyzed in downstream analysis. On the other hand, this study also significantly advances from existing studies. The data setup is more complicated than that in existing studies due to the presence of multiple datasets and, more importantly, the heterogeneity among them. Available prescreening approaches have been designed for analyzing single datasets and cannot accommodate the hetero-geneity across multiple datasets. The proposed approach is an integrative analysis approach, pools and analyzes raw data from multiple studies, and can be more effective than meta-analysis approaches. Unlike intensity approaches, the proposed approach does not need to be conducted on a case-by-case basis and does not require the full comparability of gene expression measurements from different studies. Hence it can be more broadly applicable.

## Methods

### Integrative analysis

In cancer genomic studies, among a large number of genes surveyed, only a small number of them, which are usually referred to as “susceptibility genes”, are associated with traits or clinical outcomes. The goal of prescreening is to effectively remove “noisy” genes without losing too many true positives. Only genes that pass prescreening will be analyzed in downstream analysis.

With multiple datasets, there are two distinct scenarios for their genomic basis. In this study, we focus on the scenario where multiple studies share the same biological ground, particularly the same set of susceptibility genes. An alternative scenario is where different studies have overlapping but possibly different sets of susceptibility genes. As discussed in multiple published studies [12], it is possible to carefully evaluate and select studies sharing comparable designs using the MIAME (Minimum Information About A Microarray Experiment) criteria and personal expertise [13]. In addition, for data deposited at NCBI, GEO datasets have been assembled by GEO staff using a collection of biologically and statistically comparable samples. With those selected studies, it is reasonable to expect that they share the same susceptibility genes. It is worth noting that with practical data, the identical susceptibility gene set is an *assumption*, which holds only under very cautious data selection. Prescreening under the second scenario may demand different techniques and will not be pursued in this study.

Although multiple datasets share certain common ground, the heterogeneity among them makes direct combining and analyzing inappropriate. Here the heterogeneity comes from multiple sources. First, measurements using different profiling platforms are not directly comparable. For example, one unit increase in cDNA measurement is not directly comparable to one unit increase in Affymetrix measurement. There is no guarantee that cross-platform (study) normalization always exists. In addition, other risk factors may alter the relationship between susceptibility genes and response variables. For example, for both smokers and non-smokers, gene NET1 is a susceptibility gene for lung cancer. However, the strengths of associations (magnitudes of regression coefficients) are different between the two groups.

Consider data where we can describe the relationship between the response variables and gene expressions using generalized linear models. Denote *M*(>1) as the number of independent studies. For simplicity of notation, assume that the same set of *d* genes are measured in all studies. Let *Y*^1^,…,*Y*^*M*^ be the response variables and **X**^1^,…,**X**^*M*^ be the covariates (gene expressions). In study *m*=1,…,*M*, assume

(1) $$\begin{array}{lcrr} E\left(Y^{m}|X^{m}=x^{m}\right) & = & g^{-1}\left(\overset{d}{\underset{j=0}{\sum }}\alpha_{j}^{m}x_{j}^{m}\right) & \\ & = & b^{\prime \prime }\left(\overset{d}{\underset{j=0}{\sum }}\alpha_{j}^{m}x_{j}^{m}\right). & \end{array}$$

Here, $x^{m}={\left(x_{0}^{m},x_{1}^{m},\ldots ,x_{d}^{m}\right)}^{T}$, $x_{0}^{m}\equiv 1$ is the intercept, and $\left(x_{1}^{m},\ldots ,x_{d}^{m}\right)$ are the *d*-dimensional covariates. *g* is the link function and *g*^−1^ is the inverse of *g*. $\alpha^{m}=\left(\alpha_{0}^{m},\ldots ,\alpha_{d}^{m}\right)$ is the regression coefficient. *b* is the canonical parameter, and *b*^*″*^is its derivative.

Consider an example with two independent studies and *d* gene expression measurements. Assume that only the first two genes are cancer susceptibility genes. Then the regression coefficients may look like (0.1, 0.2, 0, …, 0) and (0.05, 0.3, 0, …, 0). The regression coefficients and corresponding models have the following features. First, only the first two cancer susceptibility genes have nonzero regression coefficients (i.e., the models are sparse). Thus, identification of susceptibility genes amounts to discriminating genes with nonzero coefficients from those with zero coefficients. Second, as the two studies share the same susceptibility genes, it is reasonable to assume that the two models have the same sparsity structure. Third, to accommodate heterogeneity, the nonzero coefficients of susceptibility genes are allowed to differ across studies. This strategy shares the same spirit with the fixed-effect model method [14].

We use a superscript *\*⋆” to denote the true value of the regression coefficient. Denote ${ℳ}^{m}=\left\{j:\alpha_{j}^{m⋆}\neq \right)$$\left(0,1\leq j\leq d\right\}$ as the index set of susceptibility genes with nonzero effects in study *m*. Since we assume that different studies have the same sparsity structure, ${ℳ}^{1}=\ldots ={ℳ}^{M}={ℳ}_{⋆}$. Define the sign function as *sgn*(*a*)=1,0,−1 when *a*>0,=0,<0, respectively. Assume that sgn(***α***^1⋆^)=…=sgn(***α****M*^⋆^) and sgn(cov(*Y*^1^,**X**^1^))=…=sgn(cov(*Y*^*M*^,**X**^*M*^)). This assumption postulates that the *qualitative* properties of genes, particularly whether they are positively or negatively associated with the responses, are the same across different studies. As discussed above, the nonzero values of ***α***^*m*⋆^ are not necessarily equal for different *m*. Assume *n*_*m*_ iid observations in study *m*. The total sample size is $n=\underset{m}{\sum }n_{m}$. We standardize $X_{j}^{m}$ to have zero mean and unit variance.

It is noted that the statistical framework described above postulates linear gene effects. Such an assumption has been made in a large number of cancer genomic studies. In a few recent studies, it has been suggested that the effects of gene expressions may be nonlinear. With a single dataset, a few approaches have been developed to address nonlinear gene effects, for example, interactions of multiple genes or nonlinear effects of individual genes modeled using splines. We suspect that it is possible to extend those approaches to integrative analysis. Such an effort will be pursued in future studies.

### Integrative prescreening

In study *m* (=1…*M*), for gene *j* (=1…*d*), denote $l_{m}\left(\beta_{j0}^{m}+\beta_{j}^{m}X_{j}^{m}\right)$ as the log-likelihood function of the marginal model constructed using the intercept and the *j*^*th*^ gene. Denote ${\mathbb{P}}_{n_{m}}$ as the empirical measure. We define the marginal estimate of $\beta_{j}^{m}={\left(\beta_{j0}^{m},\beta_{j}^{m}\right)}^{T}$ as

(2) $$\begin{array}{lcrr} {\hat{\beta }}_{j}^{m}={\left({\hat{\beta }}_{j0}^{m},{\hat{\beta }}_{j}^{m}\right)}^{T}={\text{argmax}}_{\beta_{j0}^{m},\beta_{j}^{m}}l_{m}\left(\beta_{j0}^{m}+\beta_{j}^{m}X_{j}^{m}\right). & & & \end{array}$$

The set of genes that pass integrative prescreening is

(3) $$\begin{array}{lcrr} {\hat{ℳ}}_{\gamma_{n}}=\left\{j:\left|\overset{M}{\underset{m=1}{\sum }}{\hat{\beta }}_{j}^{m}/M\right|\geq \gamma_{n},1\leq j\leq d\right\}, & & & \end{array}$$

where *γ*_*n*_ is the threshold. Our simulation study suggests that integrative prescreening can reduce the dimensionality of gene expressions from *d*∼10^4^ to $|{\hat{ℳ}}_{\gamma_{n}}|∼10^{2-3}$.

We approximate the relative importance of genes in the joint model (1) using the MLEs from the marginal models. Since a marginal model consists of only one gene, the estimation can be realized rapidly using existing software and conducted in a parallel way. For a gene, with data from *M* independent studies available, we define the ranking statistic as the mean of estimated coefficients. Other possibilities include the *L*^2^ norm ${\left(\underset{m}{\sum }{\left({\hat{\beta }}_{j}^{m}\right)}^{2}\right)}^{1/2}$, the max estimates $\underset{m}{\max}|{\hat{\beta }}_{j}^{m}|$, the min estimates $\underset{m}{\min}|{\hat{\beta }}_{j}^{m}|$ and others. With the mean as the ranking statistic, the theoretical validity of integrative prescreening is rigorously established in Additional file 1. Under reasonable conditions, it can be shown that with probability going to one, all the important genes can pass the integrative prescreening with properly chosen *γ*_*n*_. It is expected that the validity of other ranking statistics can be established in a similar manner, under slightly different data assumptions. In numerical studies, we have experimented with different ranking statistics and found that the mean has the best finite-sample performance.

We note that all existing prescreening methods are for one single dataset, while the proposed integrative prescreening is applicable to multiple datasets. When there is only one single dataset, the proposed integrative prescreening boils down to the marginal likelihood prescreening proposed in [11]. That is, the contribution of the proposed method is not a prescreening utility at the level of single dataset, but how to effectively integrate prescreening utilities via combining information from multiple datasets. In this sense, the integrative prescreening with multiple datasets is *not comparable* with some existing prescreening methods for a single dataset. We suspect that other single-dataset screening methods, including those referred above, can be extended to accommodate multiple datasets. However, such an extension has not been investigated in the literature and is beyond the scope of this study.

### Operating characteristics

**Figure 1:**
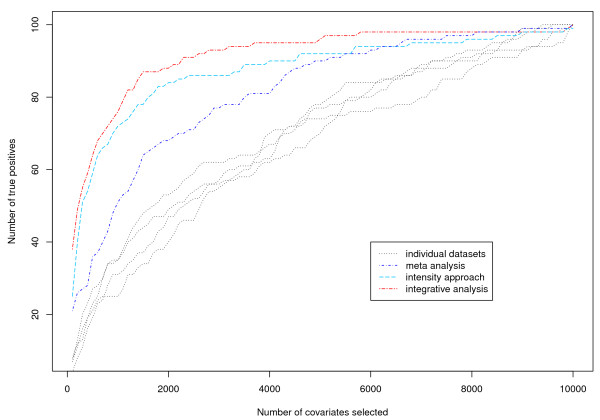
**Operating characteristics of different approaches for a simulated dataset.** Black lines: prescreening each dataset separately; Blue line: meta analysis; Light blue line: intensity approach; Red line: integrative analysis.

We simulate four datasets under the setting described in row 13 of Table 1. The simulated data consists of 10,000 genes. To better gauge performance of the proposed approach, we also consider the following alternatives. (a) Prescreening with each dataset separately; (b) An intensity approach. Since the four datasets are generated under similar settings, we adopt an intensity approach [15] and search for transformations that make gene expressions in different studies directly applicable. We then pool all four datasets together and analyze as if they were from a single study; and (c) Meta-analysis. We first analyze each dataset separately. When conducting prescreening, we effectively assign a rank to each gene. After ranking genes with each dataset separately, we compute the sums of ranks across four studies. The top ranked genes are selected. In addition, we have also considered a more “classic” meta analysis approach. For each gene in each dataset, a p-value is computed from the logistic regression. For each gene, the four p-values are then combined using the Fisher’s approach. Ranking of genes is then conducted using the overall p-values. We have compared the two meta analysis approaches and found that their performances are comparable. Only results using the first meta analysis approach are presented. Prescreening results are presented in Figure 1.

**Table 1:** Simulation: summary based on 1000 replicates

												
***n***	***#clus***	***c***	***ρ***	***In****d*_**10**_	*Int*_**10**_	*Meta*_**10**_	***T***_**5**_	***T***_**10**_	***T***_**20**_	***P***_***opt***_	***T***_***opt***_	
15	200	0.3	0.3	19	55	29	40	58	73	2200	78	
15	200	0.6	0.3	48	88	90	97	99	99	600	98	
15	200	0.3	0.6	20	56	30	43	59	74	1800	72	
15	200	0.6	0.6	50	94	91	98	98	100	400	98	
15	200	0.3	0.9	19	55	30	44	59	75	1900	72	
15	200	0.6	0.9	51	94	90	100	100	100	300	100	
15	400	0.3	0.3	19	48	31	43	58	74	3400	73	
15	400	0.6	0.3	54	87	89	97	97	97	500	94	
15	400	0.3	0.6	21	58	32	43	60	76	3700	75	
15	400	0.6	0.6	53	93	92	98	100	100	500	97	
15	400	0.3	0.9	20	57	30	45	61	78	3500	74	
15	400	0.6	0.9	53	95	93	98	99	100	700	98	
30	200	0.3	0.3	30	71	49	59	73	84	2100	86	
30	200	0.6	0.3	68	92	96	99	100	100	400	99	
30	200	0.3	0.6	31	72	50	60	74	86	1900	85	
30	200	0.6	0.6	69	96	96	99	100	100	400	99	
30	200	0.3	0.9	30	72	50	60	73	85	1900	84	
30	200	0.6	0.9	69	96	97	100	100	100	700	100	
30	400	0.3	0.3	31	68	50	62	74	85	3700	80	
30	400	0.6	0.3	70	93	96	99	100	100	400	97	
30	400	0.3	0.6	31	72	50	62	74	85	3600	82	
30	400	0.6	0.6	71	94	97	99	100	100	400	98	
30	400	0.3	0.9	32	73	49	62	75	87	3500	84	
30	400	0.6	0.9	71	96	97	100	100	100	600	99	
60	200	0.3	0.3	50	90	80	90	95	98	800	93	
60	200	0.6	0.3	92	96	100	100	100	100	900	100	
60	200	0.3	0.6	50	91	79	89	95	98	800	95	
60	200	0.6	0.6	92	96	100	100	100	100	1100	100	
60	200	0.3	0.9	50	91	80	90	96	99	2000	99	
60	200	0.6	0.9	92	96	100	100	100	100	2100	100	
60	400	0.3	0.3	50	92	80	91	95	98	800	88	
60	400	0.6	0.3	92	96	100	100	100	100	800	100	
60	400	0.3	0.6	49	89	80	90	95	98	800	89	
60	400	0.6	0.6	92	93	100	100	100	100	1000	100	
60	400	0.3	0.9	48	92	81	91	96	99	1400	93	
60	400	0.6	0.9	92	96	100	100	100	100	1600	100	

With all approaches, the number of true positives increases as the number of selected genes increases, as expected. The proposed integrative prescreening is dominatingly better, as when a fixed number of genes are selected, it has the largest number of true positives.

With the proposed approach, analysis of one replicate takes 4.7 minutes on a regular desktop PC. With the proposed approach and meta analysis, the same marginal logistic models are fit. These two approaches have different ways of combining analysis results. However, the cost of combining is negligible compared to marginal regression. Thus, they have almost identical computational cost. With intensity approaches, computational cost comes from two sources. The first is transformation of gene expressions, and the second is *d* logistic regressions with sample size $n(=\sum n_{m})$. The computational cost of logistic regression is comparable to that with the proposed approach and meta analysis. However, the computational cost of gene expression transformation may vary from a few seconds to over 30 minutes, depending on data and transformation approaches used.

### Practical considerations

With the proposed approach, gene ranking and selection is based only on the importance of genes estimated from data. For some cancer traits and clinical outcomes, there are genes or sets of genes (pathways, network modules) that have been validated to be important. In practice, the proposed approach can be modified to ensure that those genes automatically pass the prescreening.

The threshold *γ*_*n*_controls how many genes pass prescreening. In practice, there may be multiple ways of determining *γ*_*n*_. The first is to set $\left|{ℳ}_{\gamma_{n}}\right|$ equal to a prefixed number, say 2000, which may reflect researchers’ prior knowledge of the number of cancer susceptibility genes or limitation in computing power. In addition, an *ad hoc* data-dependent approach is described in the Simulation study section. Our limited simulation suggests its satisfactory performance.

Different studies may have only partially matched gene sets. Assume, for example, that gene *j* is only measured in studies 1,…,*K* with *K*<*M*. We modify the ranking statistic as $\left|\overset{K}{\underset{m=1}{\sum }}{\hat{\beta }}_{j}^{m}/K\right|$. We note that, the reliability of ranking statistic decreases as *K* decreases. In practical data analysis, we suggest an unsupervised prescreening and remove genes that are measured in only a small number of studies.

## Results and discussion

### Simulation study

We consider the following simulation settings: (a) number of independent studies M=4; (b) sample size per study *n*_*m*_=15 (30 and 60); (c) 200 (and 400) gene clusters and 50 genes per cluster. Thus, the total number of genes is d = 10,000 (and 20,000); (d) within gene clusters 1,…,10, there are 10 genes with nonzero regression coefficients. Thus, there are a total of 100 genes associated with response variables; (e) for genes with nonzero effects, we generate their regression coefficients from *Unif*[*c*,2*c*] with c=0.3 (and 0.6); (f) genes *i* and *j* within the same cluster have correlation coefficient *ρ*^|*i*−*j*|^ with *ρ*=0.3 (0.6 and 0.9). Genes in different clusters are independent. We generate the binary response variables from the logistic regression models and Bernoulli distribution.

The simulation settings closely mimic cancer genomic studies where genes have the pathway structure. Genes within the same pathways tend to have strongly correlated expressions, whereas genes within different pathways tend to have weakly correlated or independent expressions. Among a large number of pathways, only a few are associated with cancer responses. Since the pathways are not tailored to any specific response, even in an important pathway, only a subset of the genes are associated with responses.

In Table 1, we investigate the performance of integrative prescreening when 5%, 10% and 20% of the genes are selected. We use *n* to denote the sample size of each study and *#clus* to denote the number of clusters. Nonzero regression coefficients are generated from *Unif *[*c*, 2*c*]. We use *Ind*^10^, *Int*^10^, *Meta*^10^ to denote the number of true positives when the top 10% genes are selected by analyzing one single dataset, using the intensity approach, and conducting meta analysis, respectively. The numbers of true positives when the top 5%, 10%, and 20% genes are selected are denoted as *T*^5^, *T*^10^, *T*^20^. The numbers of genes selected and numbers of true positives with data-dependent *γ*_*n*_ are denoted as *P*^*opt*^ and *T*^*opt*^ respectively. Of note, the maximum number of selected genes is 4,000, which has affordable computational cost with most existing analysis approaches. As expected, when the number of selected genes increases, the number of true positives increases significantly. Under quite a few scenarios, almost all of the true positives pass prescreening. Examination of Table 1 also suggests that as sample size increases, performance of prescreening improves. As strength of signal increases, performance of prescreening improves. Moreover, as correlation increases, performance of prescreening improves. Under our simulation settings and in practical cancer genomic studies, genes associated with response variables tend to be correlated with other genes associated with responses but uncorrelated with “noisy” genes. Consider for example the extreme case where two genes associated with response have identical expressions. If each of those two genes has regression coefficient *c*, then in the marginal models, both genes would have regression coefficients 2*c*. That is, the signal is strengthened and more easily detectable.

We also consider the alternatives described in the “Operating characteristics” section. For the alternative approaches, we show the number of true positives when 10% of the genes are selected. It is clear that the intensity approach and meta-analysis have better performance than the prescreening with individual datasets. This justifies combining information across multiple heterogeneous studies. However, performances of the intensity approach and meta-analysis are worse than that of the proposed approach. Consider for example row 7 of Table 1. When 10% of the genes are selected, the numbers of true positives that pass prescreening are 19 (prescreening with individual dataset), 48 (intensity approach), 31 (meta analysis) and 58 (integrative analysis), respectively. Such observation suggests that the proposed approach is more effective in extracting useful information.

In our first set of simulation, we prefix the number of selected genes, as has been done in several published studies. Here the number of selected genes is affected more by computational limitation and prior knowledge as opposed to observed data. We have examined recent literature and found that the determination of threshold *γ*_*n*_ has not been well investigated. One approach proposed in [10] is cross validation. We have experimented with this approach and found unsatisfactory empirical performance. As [10] does not provide theoretical justification for the cross validation approach, it is not clear why it fails in integrative prescreening.

Consider the following *ad hoc* approach. We use the subscript “(*i*)” to denote the gene with the *i*^th^ largest ranking statistic. That is, $\left|\underset{m}{\sum }{\hat{\beta }}_{\left(1\right)}^{m}/M\right|\geq \left|\underset{m}{\sum }{\hat{\beta }}_{\left(2\right)}^{m}/M\right|\geq \ldots \geq \left|\underset{m}{\sum }{\hat{\beta }}_{\left(d\right)}^{m}/M\right|$, where ${\hat{\beta }}_{\left(j\right)}^{m}$ is the maximum likelihood estimator from the *j*th marginal model for the *m*th dataset, *j*=1,…,*d* and *m*=1,…,*M*. Denote $X_{\left(i\right)}^{m}$ as the gene expression corresponding to ${\hat{\beta }}_{\left(i\right)}^{m}$. Define $R_{\left(j\right)}=\overset{M}{\underset{m=1}{\sum }}l_{m}({\hat{\beta }}_{0}^{m}+{\hat{\beta }}_{\left(1\right)}^{m}X_{\left(1\right)}^{m}+\ldots +{\hat{\beta }}_{\left(j\right)}^{m}X_{\left(j\right)}^{m})$, where *l*_*m*_(·) is the log-likelihood function from the *m*th dataset. We propose determining the number of selected genes as

(4) $$\begin{array}{lcrr} P_{\mathrm{opt}} & = & \mathrm{argmi}n_{k}\left\{R_{\left(k\right)}\geq (1-\tau )\mathrm{ma}x_{j}R_{\left(j\right)}\right\}, & \end{array}$$

where *τ* is a fixed constant. In (3), *max*_*j*_*R*_(j)_ is the largest log-likelihood that can be achieved with the *d* genes. We select *P*_*opt*_ genes with which the log-likelihood is within 1−*τ*of the maximum value. This approach is intuitively reasonable. The likelihood function is expected to increase as susceptibility genes enter the models. It is expected to remain mostly unchanged as noisy genes enter the models. Thus, we seek to identify the top *P*_*opt*_ genes such that the likelihood function is sufficiently large. We note that this approach is closely related to saturated models, which usually indicate over-fitting. However, as the goal of prescreening is to preliminarily select genes, false positives and over-fitting are of less concern. Fewer genes pass prescreening as *τ* increases. In our numerical study, we set *τ*=0 and achieve the maximum value of the likelihood. In practice, to avoid an infinitesimal increase in the likelihood at the price of a dramatic increase in the number of genes, we also suggest considering *τ*=0.01 and 0.05.

**Figure 2:**
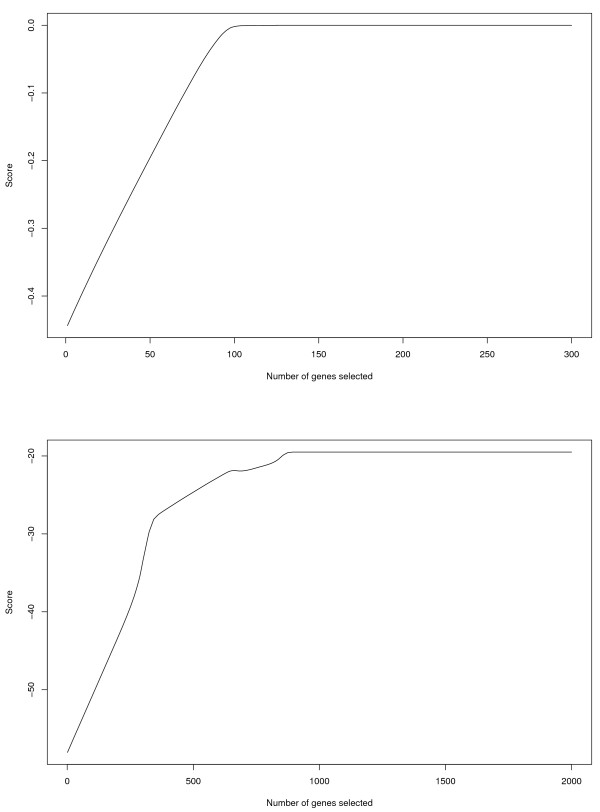
Analysis of pancreatic cancer data (upper panel) and liver cancer data (lower panel): score as a function of number of genes selected.

In Figure 2, for the pancreatic and liver cancer microarray studies described in the Data analysis section, we show *R*_(*j*)_, referred to as “score”, as a function of *j*. Similar plots are obtained for simulated datasets (omitted here). In all plots we have examined, an overall non-decreasing trend of the score is observed, and a plateau happens when a small to moderate number of genes are selected. In Table 1, we show the simulation results with *γ*_*n*_selected using the approach described above. In the computation, we search over *P*_*opt*_=100,200,300,… Under the simulated scenarios, *P*_*opt*_ is between 400 and 3700, and more than 70% of the true positives are selected. Under quite a few simulated scenarios, almost all of the true positives are selected. More simulation studies are provided in Additional file 2.

### Data analysis

#### Pancreatic cancer studies

Pancreatic ductal adenocarcinoma (PDAC) is a major cause of malignancy-related death. Apart from surgery, there is no effective therapy and even resected patients usually die within one year postoperatively. Several studies have applied microarray technology to pancreatic cancer, targeting at identifying pancreatic cancer markers. We collect and analyze the four datasets described in [16-19]. We provide brief data descriptions in Table 2. Two of the four studies use cDNA arrays, and two use oligonucleotide arrays. Cluster ID and gene names are assigned to all of the cDNA clones and Affymetrix probes based on UniGene Build 161. The two sample groups analyzed are PDAC and normal pancreatic tissues. Data preprocessing, including normalization, is carried out for each dataset separately. We identify 2,984 genes that are measured in all four studies. For Affymetrix expression measurements, we add a floor of 10 and make log2 transformations. We standardize each gene expression to have zero mean and unit variance. In Table 2, PDAC, N, Array and UG refer to the number of PDAC samples, the number of normal samples, the type of array used and the number of unique UniGene clusters respectively.

**Table 2:** Pancreatic cancer studies

				
**Dataset**	**P1**	**P2**	**P3**	**P4**
Author	Logsdon	Friess	Iacobuzio-Donahue	Crnogorac-Jurcevic
PDAC	10	8	9	8
N	5	3	8	5
Array	Affy. HuGeneFL	Affy. HuGeneFL	cDNA Stanford	cDNA Sanger
UG	5521	5521	29621	5794

#### Liver cancer studies

Gene profiling studies have been conducted on hepatocellular carcinoma (HCC), which is among the leading causes of cancer death in the world. Four microarray studies were conducted and described in [20]. We provide the data information in Table 3. Datasets D1–D4 were generated in three different hospitals in South Korea. Although the studies were conducted in a controlled setting, the researchers “failed to directly merge the data even after normalization of each dataset” [20]. In D1–D3, expressions of 10,336 genes are measured. In D4, expressions of 9,984 genes are measured. We focus on the 9,984 genes that are measured in all four studies. For each dataset, the within print-tip group normalization is carried out. For each dataset, we normalize each gene expression to have zero mean and unit variance. In Table 3, “Tumor” refers to the number of tumor samples. “Normal” refers to the number of normal samples. Numbers in the “()” are the number of subjects used in the analysis. Ver. 2 chips have different spot locations from Ver. 1 chips.

**Table 3:** Liver cancer studies


**Dataset**	**D1**	**D2**	**D3**	**D4**
Experimenter	Hospital A	Hospital B	Hospital C	Hospital C
Tumor	16 (14)	23	29	12 (10)
Normal	16 (14)	23	5	9(7)
Chip type	cDNA(Ver.1)	cDNA(Ver.1)	cDNA(Ver.1)	cDNA(Ver.2)
(Cy5:Cy3)	sample:normal liver	sample:placenta	sample:placenta	sample:sample

We conduct the proposed integrative prescreening. In Figure 2, we show the score as a function of the number of selected genes. For the pancreatic cancer studies, 117 genes pass the prescreening. For the liver cancer studies, 873 genes pass the prescreening. We also apply the alternative prescreening approaches described in the Operating characteristics section. To better compare, we match the number of selected genes using different approaches. The main observation from Table 4 is that *the sets of genes that pass different prescreenings are considerably different*. The concordance of the proposed approach with the intensity approach and meta analysis is better than that with prescreening individual datasets. This observation is reasonable, as the proposed approach, intensity approach and meta-analysis all combine information across multiple studies. However, since they use different ranking statistics, the prescreening results are significantly different with practical data.

**Table 4:** Data analysis: number of overlapped genes selected using different approaches

							
**Pancreatic cancer study**							
	*S*_1_	*S*_2_	*S*_3_	*S*_4_	Intensity	Meta	Integrative
*S*_1_	117	10	11	11	20	24	27
*S*_2_		117	6	23	35	34	36
*S*_3_			117	8	29	31	41
*S*_4_				117	37	38	43
Intensity					117	94	91
Meta						117	92
Integrative							117
**Liver cancer study**							
	*S*_1_	*S*_2_	*S*_3_	*S*_4_	Intensity	Meta	Integrative
*S*_1_	873	120	77	85	245	252	227
*S*_2_		873	114	107	398	293	223
*S*_3_			873	93	205	253	341
*S*_4_				873	167	247	382
Intensity					873	372	395
Meta						873	492
Integrative							873

Analysis of each dataset separately (*S*_1_…*S*_4_), intensity approach, meta analysis, and integrative prescreening.

Prescreening is used in cancer genomic marker discovery studies. It is worth re-emphasizing that prescreening is only step one in the discovery process. It needs to be coupled with downstream analysis. We have carefully searched literature on prescreening, however, failed to find consensus on evaluation of prescreening results in practical data analysis. The ultimate evaluation may be based on the number of true positives passing prescreening, as in simulation study. However, with our limited understanding of cancer genomics and lack of consensus on for example pancreatic and liver cancer markers, it is difficult to objectively determine the number of true positives. Evaluation based on true positives may be possible in the future when enough knowledge on cancer genomics is available.

Here we consider an approach which may provide partial evaluation of prescreening results. This approach has been adopted in several prescreening studies with single datasets. We carry out the bridge penalized estimation and marker selection with the sets of genes selected using different prescreening approaches. We then use a LOOCV (Leave One Out cross validation) approach to evaluate prediction performance. The rationale is that if one set of genes is biologically more meaningful than the others, prediction based on the bridge estimation using this gene set should be more accurate. With the pancreatic cancer datasets, the numbers of subjects mistakenly predicted are 10 (prescreening with individual datasets), 4 (intensity approach), 5 (meta analysis) and 2 (integrative prescreening), respectively, leading to prediction error rates 17.9%, 7.1%, 8.9% and 3.6%. With the liver cancer datasets, the numbers of subjects mistakenly predicted are 34 (prescreening with individual datasets), 31 (intensity approach), 29 (meta analysis) and 23 (integrative prescreening), respectively, leading to prediction error rates 27.2%, 24.8%, 23.2% and 18.4%. Such results suggest satisfactory performance of the proposed approach. It is worth noting that although the bridge penalization has been used in multiple gene expression studies, it is only one of the many available analysis approaches, and different analysis approaches have been shown to lead to different cancer markers and prediction results. Thus, the above evaluation results should be interpreted cautiously.

## Conclusions

In cancer genomic research, integrative analysis provides a cost effective way of improving reproducibility by pooling and analyzing data from multiple studies. In integrative analysis, computational difficulty can be caused by the high dimensionality of genomic measurements. In this article, we investigate integrative prescreening, which can effectively reduce the dimensionality and hence computational cost. Numerical studies demonstrate satisfactory performance of the proposed approach.

Conceptually, the proposed integrative prescreening shares similar spirit with existing prescreening approaches. The most important contribution of this study is an approach that has better finite-sample performance than existing alternatives, while having the desired statistical properties. The proposed approach allows for different nonzero regression coefficients across multiple studies for susceptibility genes, which effectively accommodates the heterogeneity across studies. In the literature, the problem of “how many genes should be selected” has not been solved. Another contribution of this study is a data-dependent stopping rule, which has satisfactory performance in numerical studies. In the analysis of real data, it is difficult to objectively evaluate the effectiveness of prescreening. We conduct an indirect, partial evaluation by investigating the prediction performance of susceptibility genes identified in downstream analysis using bridge penalization. More research is needed on the evaluation of prescreening with real data. In the analysis of single datasets, recent studies suggest that conducting prescreening iteratively may improve finite-sample performance. We expect that it is also possible to conduct the proposed integrative prescreening iteratively.

## Competing interests

The authors declare that they have no competing interests.

## Author’s contributions

All authors were involved in the study design, data analysis and writing. SM wrote the R code for data analysis. All authors read and approved the final manuscript.

## Supplementary Material

Additional file 1 **Asymptotic properties[**[11]**,**[21]**].** Click here for file

Additional file 2 **Additional simulation study **[22]**.** Click here for file
