# Supplementary material for: Integrative prescreening in analysis of multiple cancer genomic studies
Source: BMC Bioinformatics. 2012 Jul 16;13:168. doi: 10.1186/1471-2105-13-168 (PMC3436748; doi:10.1186/1471-2105-13-168)
Supplement: Additional file 1 — Asymptotic properties[[11],[21]]. [file 1471-2105-13-168-S1.pdf]

Additional File I: Asymptotic properties

We establish asymptotic properties of the proposed integrative prescreening. The asymptotic study is more complicated than that in existing studies because of the presence of multiple datasets and, more importantly, the heterogeneity among them. Note that a generalized liner model can also be written in the canonical form $f_Y(y;\theta) = \exp(y\theta - b(\theta) + c(y))$, with known $b(\theta)$ and $c(y)$ functions. Let $\boldsymbol{\beta}_j^m = (\beta_{j0}^m, \beta_j^m)^T$ denote the two-dimensional parameter and $\mathbf{X}_j^m = (1, X_j^m)^T$. We use the superscript "$\star$" to denote the true regression parameter. We make the following assumptions.

A1. For $m = 1 \dots M$, $j = 1 \dots d$, the Fisher information for the marginal regression $I_j^m(\boldsymbol{\beta}_j^{m\star}) = \mathrm{E}\left\{b''(\boldsymbol{\beta}_j^{m\star T}\mathbf{X}_j^m)\mathbf{X}_j^m\mathbf{X}_j^{mT}\right\}$ is finite and positive definite. Moreover, $\|I_j^m(\boldsymbol{\beta}_j^m)\|$ is bounded from above in a local neighborhood of $\boldsymbol{\beta}_j^{m\star}$, denoted as $\mathbf{B} = B \times B$, which is a square with the width B;

A2. $b''(\cdot)$, the second derivative of $b(\cdot)$, is continuous and positive;

A3. For all $\boldsymbol{\beta}_j^m \in \mathbf{B}$, $\mathrm{E}(l(\boldsymbol{\beta}_j^{m\star T}\mathbf{X}_j^m) - l(\boldsymbol{\beta}_j^{mT}\mathbf{X}_j^m)) \geq V_n\|\boldsymbol{\beta}_j^{m\star} - \boldsymbol{\beta}_j^m\|^2$, for some positive V_n bounded from below uniformly over $j = 1 \dots d$ and $m = 1 \dots M$;

B1. The covariates X_j^m are bounded with $\|X_j^m\|_\infty \leq K_n$ for some constant K_n;

B2. The variance $\mathrm{var}(\boldsymbol{\alpha}^{m\star T}\mathbf{X}^m)$ is bounded from above and below for $m = 1, \dots, M$.

Assumption A1 ensures the existence of the marginal MLEs. A1–A3 are satisfied in a lot of generalized linear models, particularly including linear regression, logistic regression and Poisson regression. B1 is assumed for technical convenience, although the actual value of bound may remain unknown in practice. B2 is assumed so that the models are stable.

Proposition 1. *The marginal surrogates* $\left\{S_j = 1/M\sum_{m=1}^M \beta_j^{m\star},\ j = 1, \dots, d\right\}$ *have the same sparsity structure as that of* $\left\{\sum_{m=1}^M cov(Y^m, X_j^m),\ j = 1, \dots, d\right\}$.

That is, (i) marginally unimportant genes are marginally uncorrelated with the response variables; and (ii) when genes in the nonsparsity set are marginally correlated with the response variables, i.e., $\min_{j\in\mathcal{M}_\star}|cov(Y^m, X_j^m)| \geq c_{1n}$, *for* $m = 1 \dots M$ *and some positive constant* c_{1n}, *there exists some positive constant* c_{2n} *such that*

$$\min_{j\in\mathcal{M}_\star}\Big|\sum_{m=1}^M \beta_j^{m\star}/\sum_{m=1}^M cov(Y^m, X_j^m)\Big| \geq c_{2n}. \tag{1}$$

Here we define $(\beta_{j0}^{m\star}, \beta_j^{m\star})^T = \mathrm{argmax}_{\beta_{j0}^m, \beta_j^m}\mathrm{E}l_m(\beta_{j0}^m + \beta_j^m X_j^m)$, where E is the expectation. Proposition 1 establishes the connection between the marginal surrogates $\{S_j\}$, which describe the sparsity structure of

the joint models, with the marginal correlation coefficients. Based on Proposition 1, the main properties of integrative prescreening are summarized in the following theorem.

Theorem 1. *Suppose that assumptions A1–A3 and B1–B2 hold, then*

(i) $E\sup_{j\in\mathcal{M}_\star}\left|\sum_{m=1}^{M}\hat{\beta}_j^m/M-\sum_{m=1}^{M}\beta_j^{m\star}/M\right|=O(\sqrt{\log d/n})$.

(ii) If in addition $\min_{j\in\mathcal{M}_\star}|cov(Y^m,X_j^m)|\geq c_{1n}$ *for* $m=1\ldots M$*, then by taking* $\gamma_n=c_{1n}c_{2n}/2\gg\sqrt{\log d/n}$*, with probability one,* $\mathcal{M}_\star\subset\widehat{\mathcal{M}}_{\gamma_n}$*, as* $n\to\infty$.

The validity of marginal ranking statistic for selecting susceptibility genes has been established in Proposition 1. Result (i) of Theorem 1 further establishes that the estimated marginal ranking statistics are uniformly consistent. These two results suggest that the estimated marginal ranking statistic is asymptotically valid for selecting susceptibility genes. We note that, result (i) is valid for $d=O\{\exp(n^\alpha)\}$ with $\alpha<1$. Result (ii) establishes that, when the threshold is properly chosen, integrative prescreening is consistent. Of note, prescreening is a preliminary selection. Thus, as long as false negatives are controlled, false positives are of less concern. Result (ii) provides an asymptotic order of the threshold, but it does not suggest a way of determining its actual value with finite sample data. The following result is concerned with the number of genes that can pass prescreening.

Theorem 2. *Under assumptions A1–A3 and B1-B2, for any* $\gamma_n\gg O(\sqrt{\log d/n})$*, with probability converging to one,*

$$|\widehat{\mathcal{M}}_{\gamma_n}|\leq O\{\gamma_n^{-2}\max_{m\leq M}\lambda_{\max}(\mathbf{\Sigma}^m)\},$$

where $\mathbf{\Sigma}^m=var(\mathbf{X}^m)$ *and* $\lambda_{max}(\cdot)$ *is the largest eigenvalue of a matrix.*

Theorem 2 shows that, although the consistency properties do not depend on $\mathbf{\Sigma}^m$, the number of genes that can pass prescreening is affected by the correlation structure of gene expressions. When $\gamma_n^{-2}\max_{m\leq M}\lambda_{\max}(\mathbf{\Sigma}^m)/d\to 0$, the number of selected genes is negligible compared to d.

Proof of Proposition 1 To prove the first part, it suffices to show that for $j=1\ldots d$, the sparsity set of $\sum_{m=1}^{M}\beta_j^{m\star}$ is the same as that of $\sum_{m=1}^{M}\text{cov}(Y^m,X_j^m)$. It takes three steps.

As the first step, we establish the connection between $\text{cov}(Y^m,X_j^m)$ and $\beta_j^{m\star}$. Note that for each $j=1\ldots d$, $m=1\ldots M$, $\boldsymbol{\beta}_j^{m\star}=(\beta_{j0}^{m\star},\beta_j^{m\star})^T$ is the unique minimizer of the Kullback-Leibler distance

$$\begin{aligned}I^m(\boldsymbol{\beta}_j^m) &= \text{E}\left[\log\left\{\frac{f_Y(Y^m,\boldsymbol{\alpha}^{m\star}\mathbf{X}^m)}{f_Y(Y^m,\boldsymbol{\beta}_j^{mT}\mathbf{X}_j^m)}\right\}\right]\\ &= \text{E}\left[b'(\boldsymbol{\alpha}^{\star T}\mathbf{X}^m)\left\{\boldsymbol{\alpha}^{\star T}\mathbf{X}^m-\boldsymbol{\beta}_j^{mT}\mathbf{X}_j^m\right\}-b(\boldsymbol{\alpha}^{\star T}\mathbf{X}^m)+b(\boldsymbol{\beta}_j^{mT}\mathbf{X}_j^m)\right], \qquad (2)\end{aligned}$$

where $\mathbf{X}^m = (1, X_1^m, \ldots, X_d^m)^T$ and $\mathbf{X}_j^m = (1, X_j^m)^T$. Hence it is the unique solution of the score equations

$$\mathrm{E}\left[b'(\boldsymbol{\beta}_j^{m\star T}\mathbf{X}_j^m)\mathbf{X}_j^m\right] = \mathrm{E}\left[b'(\boldsymbol{\alpha}^{\star T}\mathbf{X}^m)\mathbf{X}_j^m\right].$$

Since $\mathrm{E}X_j^m = 0$ and $\mathrm{E}(Y^m|\mathbf{X}^m) = b'(\boldsymbol{\alpha}^{\star T}\mathbf{X}^m)$, the above equation is equivalent to

$$\mathrm{cov}(Y^m, \mathbf{X}_j^m) = \mathrm{cov}(b'(\boldsymbol{\alpha}^{\star T}\mathbf{X}^m), \mathbf{X}_j^m) = \mathrm{cov}(b'(\boldsymbol{\beta}_j^{m\star T}\mathbf{X}_j^m), \mathbf{X}_j^m). \tag{3}$$

In the second step, we show that if $\sum_{m=1}^M \mathrm{cov}(Y^m, X_j^m) = 0$, then $\sum_{m=1}^M \beta_j^{m\star} = 0$. As the marginal covariances of Y^m and X_j^m have the same signs across m, $\sum_{m=1}^M \mathrm{cov}(Y^m, X_j^m) = 0$ implies that $\mathrm{cov}(Y^m, X_j^m) = 0$ for each $m = 1 \ldots M$. By (3), it further implies that

$$\mathrm{cov}(b'(\boldsymbol{\beta}_j^{m\star T}\mathbf{X}_j^m), X_j^m) = 0.$$

If $\beta_j^{m\star} \neq 0$, then by the mean value theorem, there exists some ξ such that

$$\beta_j^{m\star}\mathrm{cov}(Y^m, X_j^m) = E\Big(b'(\beta_{j0}^{m\star} + \beta_j^{m\star}X_j^m) - b'(\beta_{j0}^{m\star})\Big)X_j^m\beta_j^{m\star} = E(b''(\xi)X_j^{m2}\beta_j^{m\star 2}) > 0. \tag{4}$$

This can not happen, since $\mathrm{cov}(Y^m, X_j^m) = 0$. Therefore it forces $\beta_j^{m\star} = 0$ for all $m = 1 \ldots M$. This implies that $\sum_{m=1}^M \beta_j^{m\star} = 0$.

In the third step, we show that if $\sum_{m=1}^M \mathrm{cov}(Y^m, X_j^m) \neq 0$, then $\sum_{m=1}^M \beta_j^{m\star} \neq 0$. Without loss of generality, we consider the case when $\sum_{m=1}^M \mathrm{cov}(Y^m, X_j^m) > 0$. As the marginal covariances of Y^m and X_j^m are of the same direction across m, this implies that $\mathrm{cov}(Y^m, X_j^m) > 0$ for all $m = 1 \ldots M$. It thus follows from (4) that $\beta_j^{m\star} > 0$ for all $m = 1 \ldots M$, which implies that $\sum_{m=1}^M \beta_j^{m\star} > 0$. The first part of the result is thus proved.

For the second part, since $|\sum_{m=1}^M \mathrm{cov}(Y^m, X_j^m)| \geq c_{1n}$ for $j \in \mathcal{M}_\star$, without loss of generality, we assume that for all $m = 1, \ldots, M$, $\mathrm{cov}(Y^m, X_j^m)$ are positive for $j \in \mathcal{M}_\star$. It follows from the score equation that

$$\sum_{m=1}^M \mathrm{E}(b'(\beta_{j0}^{m\star} + \beta_j^{m\star}X_j^m)X_j^m \geq c_{1n}, \tag{5}$$

for $j \in \mathcal{M}_\star$. By Taylor expansion, there exists $D_1 = \sup_x b''(x)$ such that

$$|b'(\beta_0^{m\star} + \beta_j^{m\star}X_j^m)X_j^m - b'(\beta_0^{m\star})X_j^m| \leq D_1\beta_j^{m\star}X_j^{m2}. \tag{6}$$

By taking expectations on both sides of (6) and the Triangle inequality,

$$|\mathrm{E}b'(\beta_{j0}^{m\star} + \beta_j^{m\star}X_j^m)X_j^m - \mathrm{E}b'(\beta_{j0}^{m\star})X_j^m| \leq D_1\beta_j^{m\star}. \tag{7}$$

It follows from (7) and the Triangle inequality that

$$\mathrm{E}\sum_{m=1}^{M} b'(\beta_{j0}^{m\star}+\beta_j^{m\star}X_j^m)X_j^m \le D_1\sum_{m=1}^{M}\beta_j^{m\star}. \tag{8}$$

Combining (5) and (8), we get

$$\min_{j\in\mathcal{M}_\star}\sum_{m=1}^{M}\beta_j^{m\star}\ge c_{3n}, \tag{9}$$

where $c_{3n}=c_{1n}D_1^{-1}$. The second assertion thus holds by letting $c_{2n}=D_1^{-1}$. □

To prove Theorem 1, we will use the following results.

Lemma 2.2.1 in [21]. Let X be a random variable with $P(|X|>x)\le Ke^{-Cx^p}$ for every $x>0$ and constants K, C and $p\ge 1$. Then $\|X\|_{\psi_p}\le((1+K)/C)^{1/p}$. Here $\|\cdot\|_{\psi_p}$ is the Orlicz norm: $\|X\|_\psi = \inf\left\{C>0: E\psi\left(\frac{|X|}{C}\right)\le 1\right\}$ and $\psi_p(x)=e^{x^p}-1$ for $p\ge 1$.

Lemma 2.2.2 in [21]. Let ψ be a convex, nondecreasing, nonzero function with $\psi(0)=0$ and $\limsup_{x,y\to\infty}\psi(x)\psi(y)/\psi(cxy)<\infty$ for a finite constant c. Then, for any random variables $X_1,\ldots,X_m$, $\|\max_{1\le j\le m}X_i\|_\psi\le K\psi^{-1}(m)\times\max_{1\le i\le m}\|X_i\|_\psi$, for a constant K depending only on ψ.

Proof of Theorem 1 An application of Theorem 1 of [11] yields that, for any $t>0$,

$$P\left(\sqrt{n}|\hat{\beta}_j^m-\beta_j^{m\star}|\ge 16k_n(1+t)/V_n\right)\le\exp(-2t^2/K_n^2), \tag{10}$$

where $k_n=b'(K_nB+B)$. Define $W_{j,n}^m=\sqrt{n}|\hat{\beta}_j^m-\beta_j^{m\star}|-16k_n/V_n$. Since for arbitrary x and positive scalers a,t, $|x-a|>t$ implies $|x|>a+t$, (10) implies that

$$P\left(|W_{j,n}^m|\ge 16k_nt/V_n\right)\le\exp(-2t^2/K_n^2).$$

It can be further expressed as

$$P\left(|W_{j,n}^m|\ge x\right)\le\exp(-Cx^2), \tag{11}$$

where $C=V_n^2/(128k_n^2K_n^2)$. Using Lemma 2.2.1 above, we obtain

$$\|W_{j,n}^m\|_{\psi_2}\le\sqrt{3C},$$

for all $1\le j\le d$ and $1\le m\le M$. Then by Lemma 2.2.2 above combined with the fact that $\limsup_{x,y\to\infty}\psi_2(x)\psi_2(y)/\psi_2(xy)=0$, there exists a universal constant $K<\infty$ with

$$\|\max_{1\le j\le d}W_{j,n}^m\|_{\psi_2}\le K\sqrt{\log(1+d)}\sqrt{3C}.$$

Since $\log(p+1) \leq 2\log p$ for all $p \geq 2$ and $\|X\|_p \leq \|X\|_{\psi_p}$ for any random variable X, it follows that

$$\mathrm{E}\max_{j=1,\ldots,d}\left|\hat{\beta}_j^m - \beta_j^{m\star}\right| \leq D_2\sqrt{\log d/n},$$

where $D_2 = 2K\sqrt{3C}$. Part (i) thus follows from the fact that

$$\left|1/M\sum_{m=1}^{M}(\hat{\beta}_j^m - \beta_j^{m\star})\right| \leq 1/M\sum_{i=1}^{M}\left|\hat{\beta}_j^m - \beta_j^{m\star}\right|. \tag{12}$$

To prove part (ii), we note that by the Markov inequality and the result from part (i),

$$P\Big(\max_{j\leq d}\Big|1/M\sum_{m=1}^{M}(\hat{\beta}_j^m - \beta_j^{m\star})\Big| \geq 1/2c_{1n}c_{2n}\Big) \leq \mathrm{E}\max_{j\leq d}\Big|1/M\sum_{m=1}^{M}(\hat{\beta}_j^m - \beta_j^{m\star})\Big|/\gamma_n \to 0.$$

Therefore with probability converging to one,

$$\min_{j\in\mathcal{M}_\star}|\sum_{m=1}^{M}\hat{\beta}_j^m| \geq \gamma_n,$$

That is, $P(\mathcal{M}_\star \in \hat{\mathcal{M}}_{\gamma_n}) \to 1$.

Proof of Theorem 2 The proof takes two steps. In the first step, we show that the size of the set: $\mathcal{M}_{\gamma_n} = \{j : |1/M\sum_{m=1}^{M}\beta_j^{m\star}| \geq \gamma_n\}$ is no greater than $O(1/M\gamma_n^{-2}\max_{m\leq M}\lambda_{\max}(\boldsymbol{\Sigma}^m))$. In the second step, we show that the size of $\widehat{\mathcal{M}}_{\gamma_n}$ is of the same order as that of $\mathcal{M}_{\gamma_n}$.

To show the first assertion, it suffices to show the Euclidean norm of $\boldsymbol{\beta}^{m\star} = (\beta_1^{m\star},\ldots,\beta_d^{m\star})^T$ is bounded by $O\{\sum_{m=1}^{M}\lambda_{\max}(\boldsymbol{\Sigma}^m)\}$. That is,

$$\|1/M\sum_{m=1}^{M}\boldsymbol{\beta}^{m\star}\|^2 \leq O\{1/M\sum_{m=1}^{M}\lambda_{\max}(\boldsymbol{\Sigma}^m)\}. \tag{13}$$

Since

$$\|1/M\sum_{m=1}^{M}\boldsymbol{\beta}^{m\star}\|^2 \leq 1/M\sum_{m=1}^{M}\|\boldsymbol{\beta}^{m\star}\|^2, \tag{14}$$

we first bound $\sum_{m=1}^{M}\beta_j^{m\star 2}$, the jth entry of the right hand side of (14). Since $b'(\cdot)$ is monotonically increasing, the function

$$\sum_{m=1}^{M}\{b'(\beta_{j0}^{m\star} + X_j^m\beta_j^{m\star}) - b'(\beta_{j0}^{m\star})\}X_j^m\beta_j^{m\star}$$

is always positive. By Taylor's expansion, we have

$$\sum_{m=1}^{M}\{b'(\beta_{j0}^{m\star} + X_j^m\beta_j^{m\star}) - b'(\beta_{j0}^{m\star})\}\beta_j^{m\star}X_j^m \geq \sum_{m=1}^{M}D_3(\beta_j^{m\star}X_j^m)^2,$$

where $D_3 = \inf_x b''(x)$, since $(\beta_{j0}^{m\star}, \beta_j^{m\star})$ is an interior point of the square $\mathbf{B}$. By taking the expectation on both sides and using $\mathrm{E}X_j^m = 0$, we have

$$\sum_{m=1}^{M} \mathrm{E}b'(\beta_{j0}^{m\star} + X_j^m \beta_j^{m\star})\beta_j^{m\star} X_j^m \geq D_3 \sum_{m=1}^{M} \mathrm{E}(\beta_j^{m\star} X_j^m)^2.$$

It follows from the score equation that

$$\sum_{m=1}^{M} \beta_j^{m\star 2} \leq D_4 \sum_{m=1}^{M} \mathrm{E}b'(\mathbf{X}^{mT}\boldsymbol{\alpha}^{m\star})X_j^m, \tag{15}$$

for some $D_4 > 0$. We further bound from above the right hand side of (15) by using $\mathrm{var}(\mathbf{X}^{mT}\boldsymbol{\alpha}^{m\star}) = O(1)$ uniformly over m. By Taylor expansion,

$$\{b'(\mathbf{X}^{mT}\boldsymbol{\alpha}^{m\star}) - b'(\alpha_0^{m\star})\}X_j^m \leq D_5 \left| X_j^m \mathbf{X}_d^{mT} \boldsymbol{\alpha}_d^{m\star} \right|,$$

where $\mathbf{X}_d^m = (X_1^m, \cdots, X_d^m)^T$ and $\boldsymbol{\alpha}_d^{m\star} = (\alpha_1^{m\star}, \ldots, \alpha_d^{m\star})^T$. By putting the above equation into the vector form and taking the expectation on both sides and sum over m, we have

$$\begin{aligned} \sum_{m=1}^{M} \left\| \mathrm{E}\{b'(\mathbf{X}^{mT}\boldsymbol{\alpha}^{m\star}) - b'(\alpha_0^{m\star})\}\mathbf{X}_d^m \right\|^2 &\leq D_5^2 \sum_{m=1}^{M} \left\| \mathrm{E}\mathbf{X}_d^m \mathbf{X}_d^{mT} \boldsymbol{\alpha}_d^{m\star} \right\|^2 \qquad (16) \\ &\leq D_5^2 \sum_{m=1}^{M} \lambda_{\max}(\boldsymbol{\Sigma}^m) \left\| \boldsymbol{\Sigma}^{m1/2} \boldsymbol{\alpha}^{m\star} \right\|^2. \end{aligned}$$

Using $\mathrm{E}\mathbf{X}_d^m = 0$ and $\mathrm{var}(\mathbf{X}^{mT}\boldsymbol{\alpha}^{m\star}) = O(1)$, we conclude that

$$\sum_{m=1}^{M} \left\| \mathrm{E}b'(\mathbf{X}^{mT}\boldsymbol{\alpha}^{m\star})\mathbf{X}_d^m \right\|^2 \leq O(\sum_{m=1}^{M} \lambda_{\max}(\boldsymbol{\Sigma}^m)) = D_6 \max_{m \leq M} \lambda_{\max}(\boldsymbol{\Sigma}^m),$$

for some positive constant D_6. This together with (15) entail (13).

To show the second step, we note that from the first step, the number of $\{j : |1/M \sum_{m=1}^{M} \beta_j^{m\star}| > \gamma_n\}$ can not exceed $O\{\gamma_n^{-2} \max_{m \leq M} \lambda_{\max}(\boldsymbol{\Sigma}^m)\}$. Thus, on the set

$$B_n = \left\{ \max_{1 \leq j \leq d} 1/M \left| \sum_{m=1}^{M} \hat{\beta}_j^m - \sum_{m=1}^{M} \beta_j^{m\star} \right| \leq \gamma_n \right\},$$

the number of $\{j : 1/M | \sum_{m=1}^{M} \hat{\beta}_j^m | > 2\gamma_n\}$ can not exceed the number of $\{j : 1/M | \sum_{m=1}^{M} \beta_j^{m\star} | > \gamma_n\}$, which is bounded by $O\{\gamma_n^{-2} \max_{m \leq M} \lambda_{\max}(\boldsymbol{\Sigma}^m)\}$. The desired result thus follows from part (ii) of Theorem 1 and the fact that $\gamma_n = c_{1n}c_{2n}/2$.
