# Supplementary material for: Integrative prescreening in analysis of multiple cancer genomic studies
Source: BMC Bioinformatics. 2012 Jul 16;13:168. doi: 10.1186/1471-2105-13-168 (PMC3436748; doi:10.1186/1471-2105-13-168)
Supplement: Additional file 2 — Additional simulation study [22]. [file 1471-2105-13-168-S2.pdf]

Additional File II: Additional simulation study

In this study, we have focused on cancer diagnosis studies where the response variables are binary. The theoretical properties are rigorously presented in Additional File I. Following a similar strategy, it is possible to extend the proposed approach to the integrative analysis of other types of response variables.

In particular, consider prognosis studies with censored survival responses. Use notations similar to those described above. In study m, denote T^m as the event time. Assume the accelerated failure time (AFT) model, where

$$\log(T^m) = \alpha_0^m + \sum_{j=1}^{d} \alpha_j^m x_j^m.$$

In [22] and references therein, the AFT model has been adopted for the integrative analysis of cancer prognosis studies with gene expression measurements. Denote C^m as the censoring time in study m. Then the observed response consists of $(\min(T^m, C^m), I(T^m \leq C^m))$. In terms of methodology, the proposed approach can be almost directly extended to accommodate censored survival response and AFT model. For estimation with the marginal models and so calculation of the ranking statistics, we adopt the weighted least squares approach described in [22].

We conduct a simulation study to evaluate integrative prescreening with censored survival data. Gene expressions are simulated in the same manner as in the "third block" of Table 1. We set the intercepts $\alpha_0^m = 0$. α_j^ms are generated in the same manner as described above. The event time T^m is generated from the AFT model. The censoring time is generated independently from an exponential distribution. The parameter of exponential distribution is adjusted so that the censoring rate is about 40%. Summary simulation results based on 1000 replicates are shown in Table 5. In this table, $\#clus$ is denoted as the number of clusters. Nonzero regression coefficients are generated from uniform distribution on $[c, 2c]$. The number of true positives when the top 10% genes are selected by analyzing one single dataset, using the intensity approach, and conducting meta analysis are denoted as Ind_{10}, Int_{10} and $Meta_{10}$ respectively. The number of true positives when the top 5%, 10%, and 20% genes are selected are denoted as T_5, T_{10} and T_{20} respectively. The numbers of genes selected and number of true positives with data-dependent γ_n are denoted as P_{opt} and T_{opt} respectively. We can see that although there are some quantitative differences, the main properties are similar to those observed in Table 1. Particularly, the proposed integrative prescreening outperforms prescreening with individual datasets, intensity approach and meta-analysis.

We expect theoretical properties similar to those described in Additional File I to hold. However, as has been shown in many published studies, theoretical properties with high-dimensional data analysis methods

need to be established on a case-by-case basis. Theoretical investigation of prescreening with censored survival data, although interesting, is beyond scope of this study.

Table 5: Simulation with censored survival response and AFT model: summary based on 1000 replicates.

n	$\#clus$	c	ρ	Ind_{10}	Int_{10}	$Meta_{10}$	T_5	T_{10}	T_{20}	P_{opt}	T_{opt}
30	200	0.3	0.3	41	59	47	53	69	81	1900	79
30	200	0.6	0.3	71	90	93	94	96	97	800	97
30	200	0.3	0.6	39	70	52	66	72	83	2200	84
30	200	0.6	0.6	63	92	93	93	94	96	500	93
30	200	0.3	0.9	28	70	52	55	70	79	2300	80
30	200	0.6	0.9	63	94	95	96	96	97	400	95
